# Supplementary material for: Structural insights into the BRAF monomer-to-dimer transition mediated by RAS binding
Source: Nat Commun. 2022 Jan 25;13:486. doi: 10.1038/s41467-022-28084-3 (PMC8789793; doi:10.1038/s41467-022-28084-3)
Supplement: Supplementary file 1 — Supplementary Information [file 41467_2022_28084_MOESM1_ESM.pdf]

Supplementary Information

Structural insights into the BRAF monomer-to-dimer transition mediated by RAS binding

Juliana A. Martinez Fiesco[1]^, David E. Durrant[2]^, Deborah K. Morrison[2*]
and Ping Zhang[1*]

[1]Center for Structural Biology and [2]Laboratory of Cell and Developmental Signaling, Center for Cancer Research, National Cancer Institute-Frederick, Frederick, MD 21702

^ These authors contributed equally to this work

* corresponding authors: morrisod@mail.nih.gov and ping.zhang@nih.gov

Supplementary Information:

Supplementary Fig. 1-6
Supplementary Tables 1-4
Supplementary References

Supplementary Figures

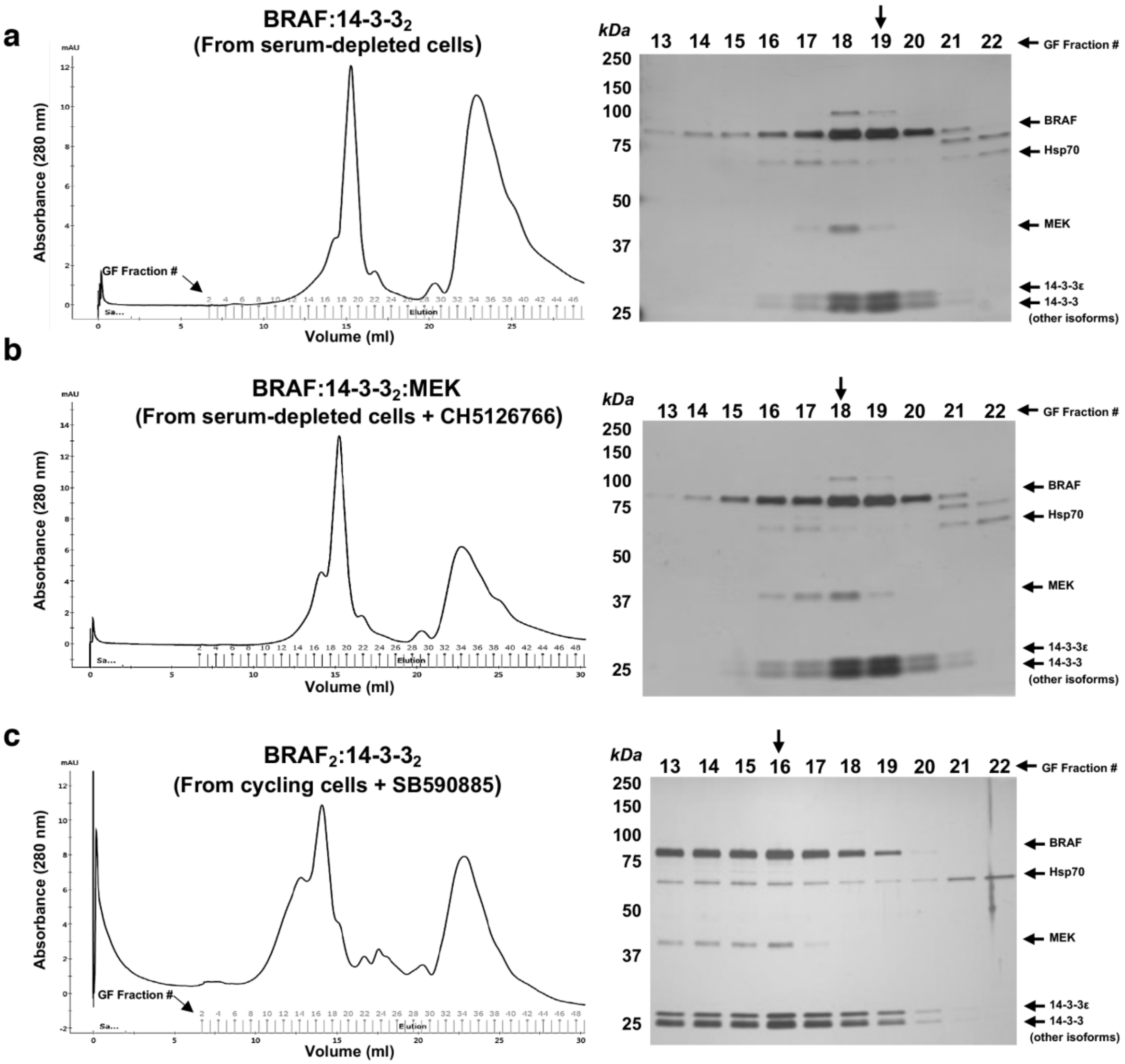

d

Protein	Accession	BRAF:14-3-3$_2$:MEK	BRAF:14-3-3$_2$	BRAF$_2$:14-3-3$_2$
14-3-3 protein epsilon	P62258	49.2	51.2	43.5
14-3-3 protein zeta/delta	P63104	23.4	24.7	25.8
14-3-3 protein beta/alpha	P31946	8.5	8.1	9.5
14-3-3 protein eta	Q04917	6.1	6.4	9.6
14-3-3 protein gamma	P61981	8.5	5.9	3.9
14-3-3 protein theta	P27348	4.3	3.6	7
14-3-3 protein sigma	P31947	0.2	0.1	0.7
MEK1	Q02750	53.6		
MEK2	P36507	46.4		

Values represent percentage of total peptides for 14-3-3 or MEK

Supplementary Fig. 1. Isolation and characterization of mammalian BRAF complexes

BRAF:14-3-3_2 (**a**), BRAF:14-3-3_2:MEK (**b**), and $BRAF_2$:14-3-3_2 (**c**) complexes were analyzed by gel filtration (GF) (left) and SDS-PAGE/silver staining (right) . The arrows indicate the sample fractions used for cryo-EM studies. Quantification of the silver stained band densities in the sample fractions used for cryoEM studies indicates a 1:1 ratio for BRAF and the 14-3-3 dimer (with all 14-3-3 isoforms pooled). The ratio of BRAF and MEK in Fraction 19 of (**a**) was 1:0.04, in fraction 18 of (**b**) was 1:0.39, and in fraction 16 of (**c**) was 1:0.18. **d** Mass spectrometry analysis of the complexes isolated in **a**-**c**.

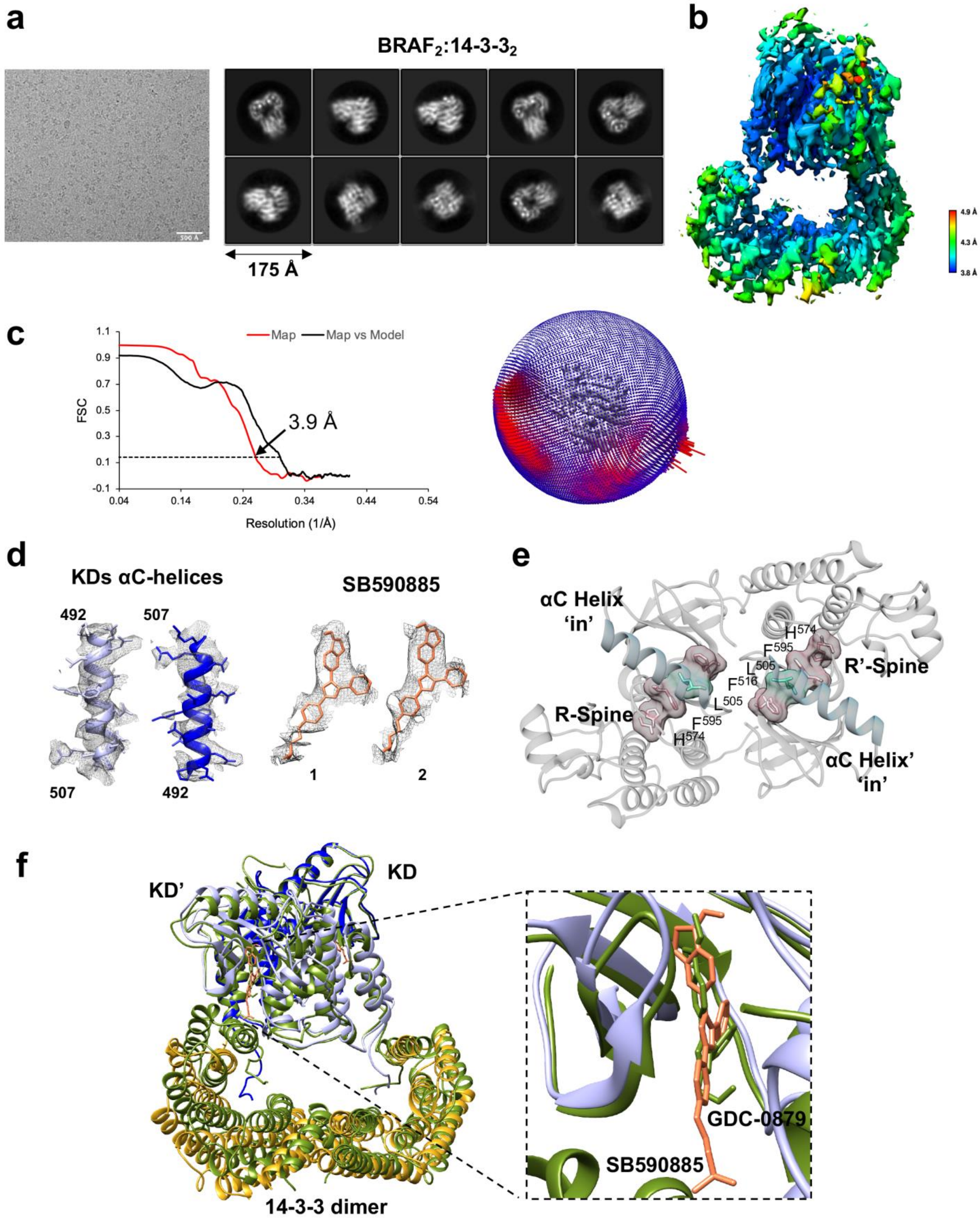
a
BRAF2:14-3-32
500 Å
175 Å
b
4.9 Å
4.3 Å
3.8 Å
c
Map
Map vs Model
FSC
3.9 Å
Resolution (1/Å)
d
KDs αC-helices
492
507
507
492
SB590885
1
2
e
αC Helix 'in'
R'-Spine
R-Spine
αC Helix' 'in'
H574
F595
L506
F516
L505
F595
H574
f
KD'
KD
14-3-3 dimer
GDC-0879
SB590885

Supplementary Fig. 2. Cryo-EM analysis of the $BRAF_2$:14-3-3_2 dimer complex

a Representative cryo-EM image of 2,998 micrographs and 2D class averages of $BRAF_2$:14-3-3_2 particles. **b** $BRAF_2$:14-3-3_2 cryo-EM map colored according to local resolution. **c** FSC curves of the cryo-EM density map (left). Reported resolutions were based on the FSC=0.143 criteria. Red curve is the $BRAF_2$:14-3-3_2 half map and black curve is the model versus map FSC. Euler angle distribution of particles used for the global reconstruction map (right). **d** Cryo-EM densities corresponding to the αC-helix and the bound molecule of SB590885 in each KD protomer. **e** Conformation of the KDs in the liganded $BRAF_2$:14-3-3_2 complex. The KD is shown in gray with the αC-helix (blue) in the active 'in' position and the R-spine residues (pink) aligned. **f** Superposition of the SB590885-liganded $BRAF_2$:14-3-3_2 structure (colored as in Fig. 1) with the crystal structure of (GDC-0879)-bound BRAF KD_2 (residues D432-R735):14-3-3_2 (in green, PDB ID: 6U2H) is shown, with an overall Cα RMSD of 2.29 Å. The inset shows the SB590885 and GDC-0879 ligands occupy overlapping positions in the active site.

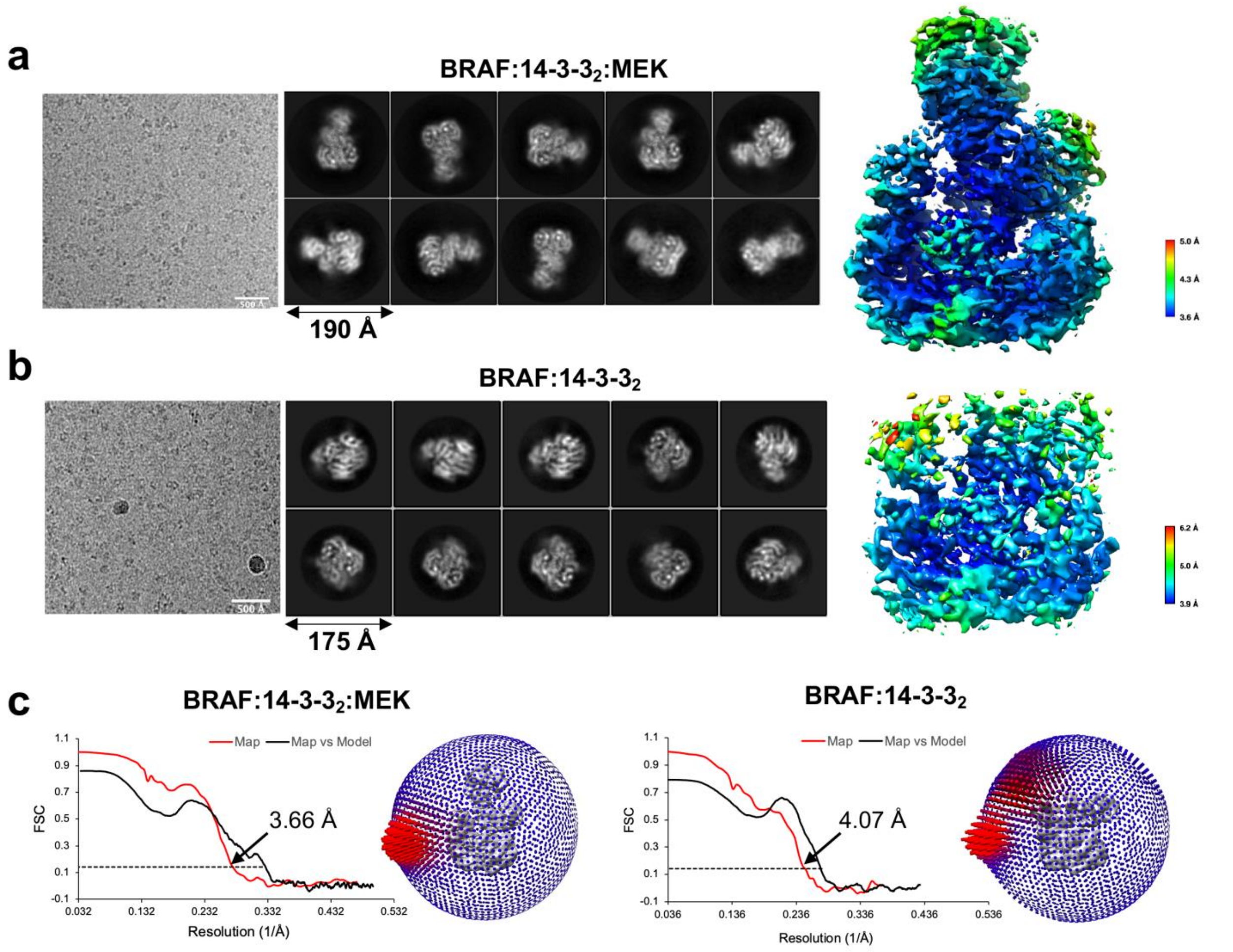

Supplementary Fig. 3. Cryo-EM analysis of autoinhibited BRAF:14-3-3$_2$:MEK and BRAF:14-3-3$_2$ complexes

a and **b** Representative cryo-EM image and 2D class averages (left) and the cryo-EM map colored according to local resolution (right) of the BRAF:14-3-3$_2$:MEK (**a**) and BRAF:14-3-3$_2$ (**b**) complexes. The complete cryo-EM data set consisted of 3,976 and 5,995 micrographs for BRAF:14-3-3$_2$:MEK and BRAF:14-3-3$_2$, respectively. **c** FSC curves of the cryo-EM density map and euler angle distribution of particles used for the global reconstruction map of BRAF:14-3-3$_2$:MEK (left) and BRAF:14-3-3$_2$ (right).

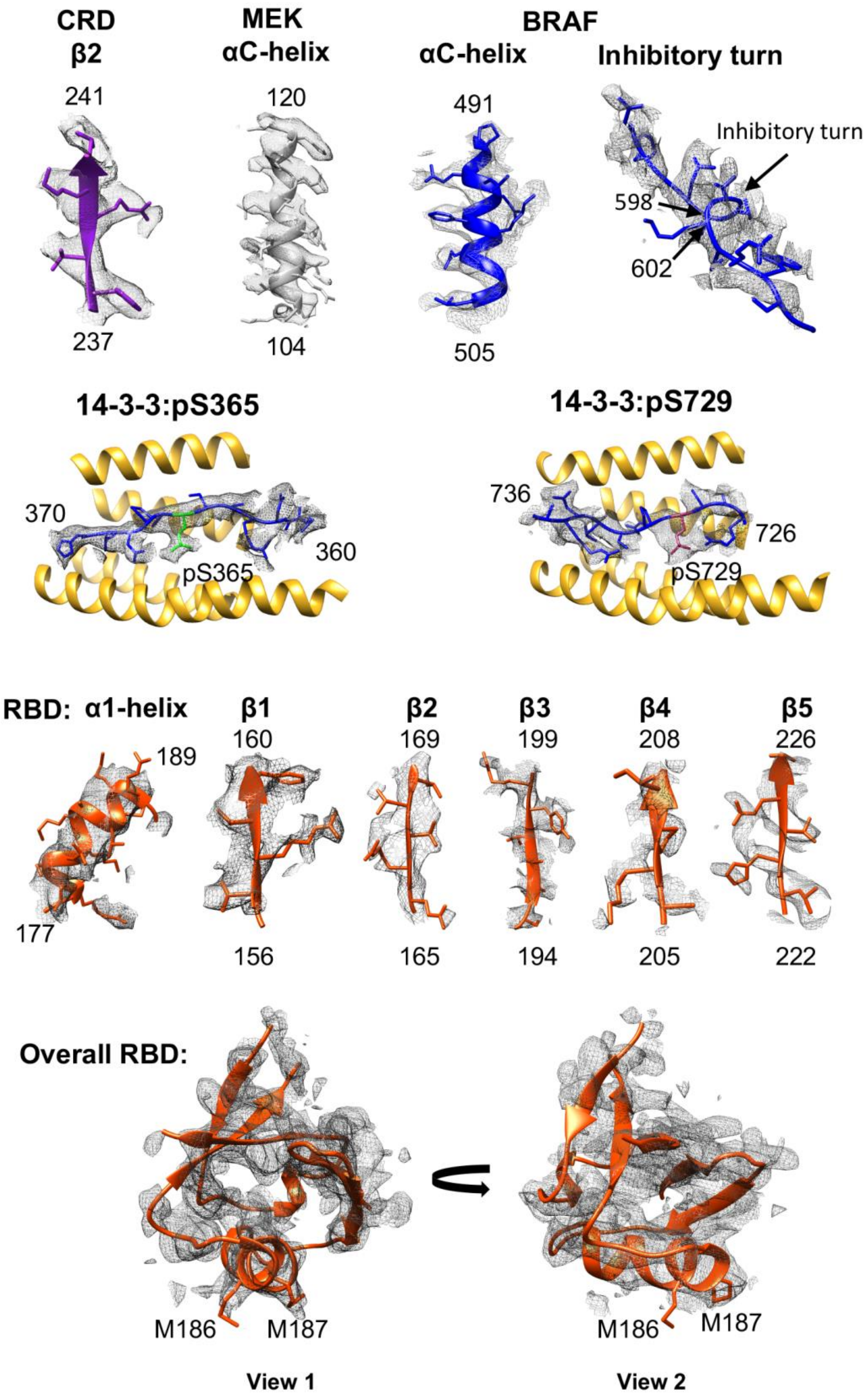

Supplementary Fig. 4. Structural analysis of the BRAF:14-3-3$_2$:MEK complex

Density maps of specific regions of BRAF (RBD, CRD, and KD), MEK, and 14-3-3 proteins are shown. Also shown, are density maps of the overall RBD, with methione residues 186 and 187 indicated.

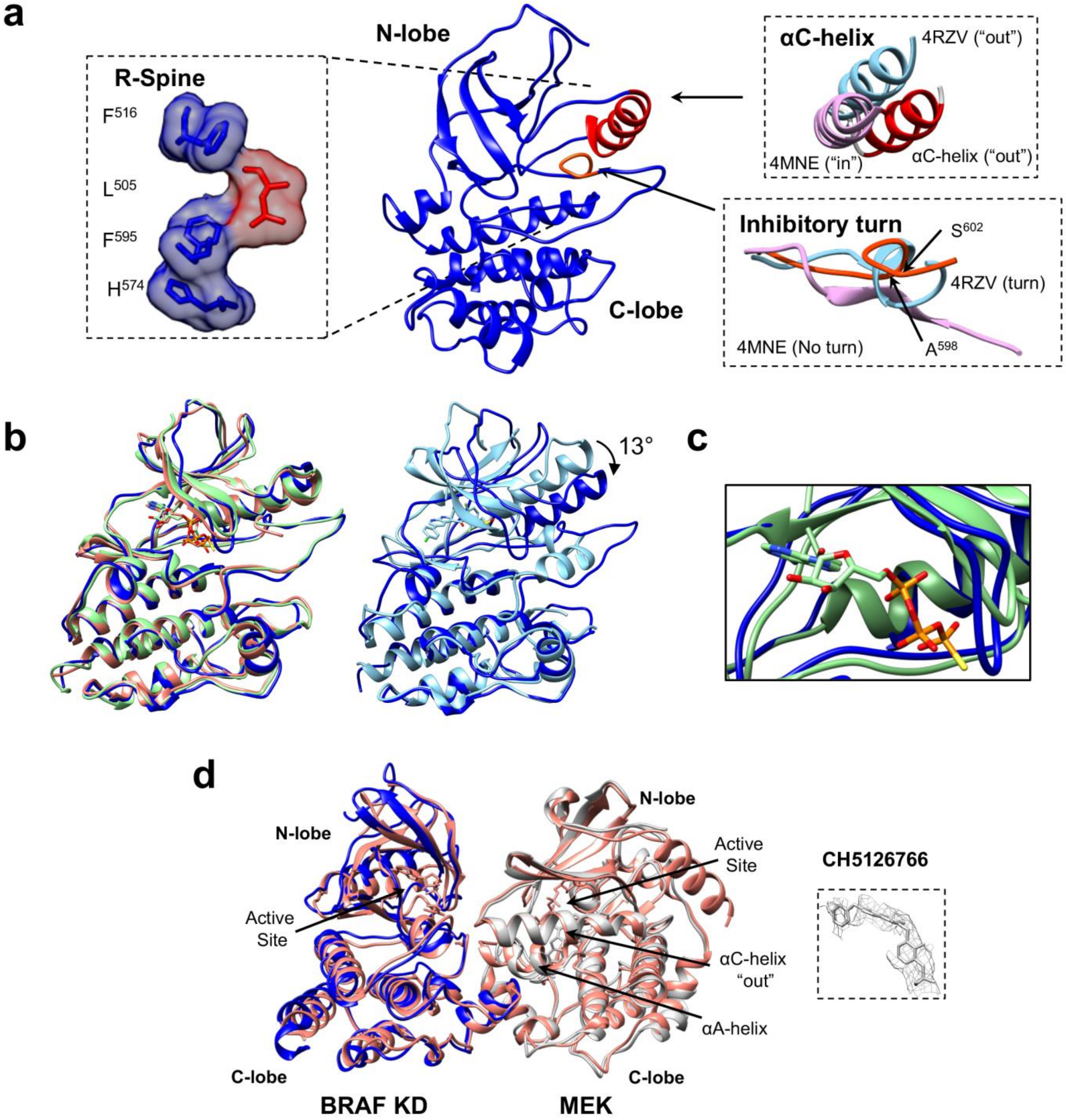

Supplementary Fig. 5. Structural analysis of the BRAF KD in the autoinhibited BRAF:14-3-3$_2$:MEK complex

a The BRAF KD in the BRAF:14-3-3$_2$:MEK complex assumes the inactive conformation, with the aC-helix in the “out” position and the R-spine broken (left inset). Insets on the right show the comparison of specific regions of the present KD structure with published BRAF KD structures in the active (PDB ID 4MNE, in pink) and inactive, inhibitory-bound (PDB ID: 4RZV,

bound to the RAF inhibitor vemurafenib, in blue) conformations. **b** (left) Superimposition of the BRAF KD (blue) in the present structure with BRAF KDs of the previously reported cryo-EM BRAF(ATP-γS):14-3-3_2:MEK1 structure (with a Cα R.M.S.D. of 0.90 Å, PDB ID:6NYB, green), and the BRAF KD(AMP-PCP):MEK1 structure (with a Cα R.M.S.D. of 0.82 Å, PDB ID: 6U2G, salmon), demonstrating the compact configuration of the N- and C-lobes in these inactive KD structures. (right) Superimposition of the BRAF KD (blue) in the present structure with the vemurafenib-bound BRAF KD structure (with a Cα R.M.S.D. of 0.84 Å, PDB ID: 4RZV, light blue), which is an example of RAF inhibitor-bound BRAF KD structures exhibiting a more open N- and C-lobe configuration. Superimposition of these structures was done based on the C-lobe of the KD. **c** Superimposition of the ATP binding pockets in the present BRAF:14-3-3_2:MEK structure (blue) and the previously reported cryo-EM BRAF:14-3-3_2:MEK1 structure bound to ATP-γS (PDB ID:6NYB, green). **d** Superimposition of the BRAF KD:MEK component of the BRAF:14-3-3_2:MEK in the present structure (BRAF KD in blue and MEK in gray) with the previously reported crystal structure of the isolated BRAF KD:MEK1 dimer (PDB ID: 6U2G, pink), with a Cα R.M.S.D. of 0.97 Å. Superimposition of these structures was done based on the BRAF KD:MEK component of the structures. Also shown, is the cryo-EM density corresponding to the MEK inhibitor CH5126766 bound to the allosteric site of MEK.

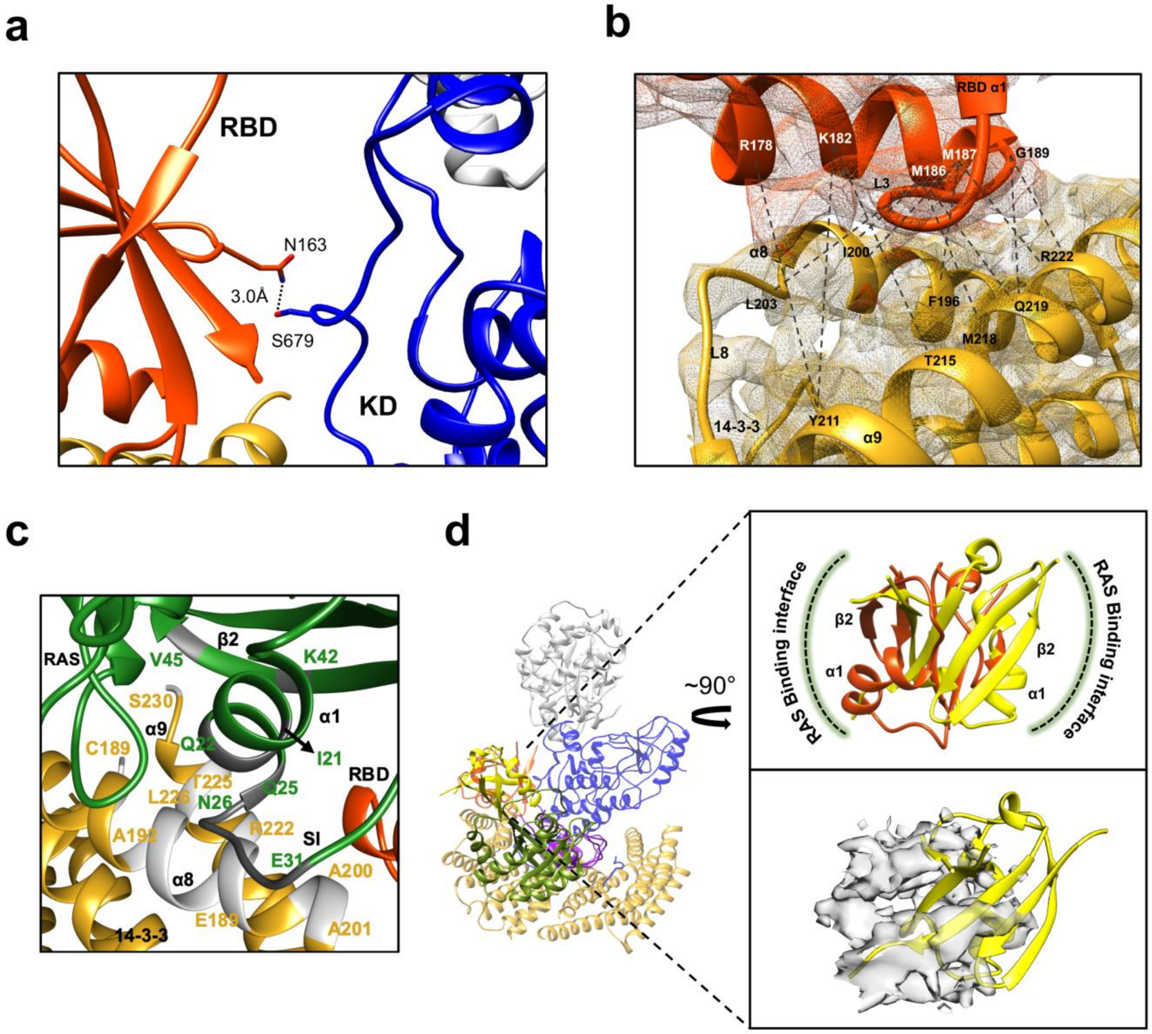

Supplementary Fig. 6 Structural analysis of BRAF RBD interactions

a Potential interaction between N163 in the BRAF RBD (red orange) and S679 in the BRAF KD (blue). **b** Density map showing the RBD:14-3-3 interface, with the RBD region colored in orange and the 14-3-3 region colored in gold. The potential interacting residues are labeled **c** Potential steric clash at the RBD:14-3-3 interface between RAS residues I21-Q22 (α1-helix), Q25-E31 (SI region), K42 and V45 (β2 sheet) with 14-3-3 α8-helix residues C189, A192-E198, I200-A201 and α9-helix residues R222, L225-T226 and S230 upon superimposition of KRAS onto the autoinhibited BRAF:14-3-3$_2$:MEK complex. RAS is colored in green, 14-3-3 in gold, and the potential clashing residues in RAS and 14-3-3 are colored in dark and light gray respectively. **d** Rigid body superposition of the BRAF:14-3-3$_2$:MEK complex (colored as in Fig.

2) and the KRAS:CRAF_RBD-CRD complexes (KRAS in green, CRAF RBD in yellow, and CRAF CRD in violet. PDB ID: 6XI7), with the alignment based on the CRD. Insets show the difference in the RBD orientation (top) and position (bottom) between the two structures. For the RBD position, the cryo-EM density map in the region of the BRAF RBD is shown in light gray and the CRAF RBD ribbon structure is in yellow.

Supplementary Tables

Supplementary Table 1. Fit of BRAF:14-3-3_2 and BRAF:14-3-3_2:MEK structures to their corresponding cryo-EM maps showing the inctive conformation of the KD is adopted.

**Structural element**	**Structural fit of BRAF:14-3-3_2 (PDB ID 7MFE) to corresponding cryo-EM density map (EMD-23814)**	**Structure fit of BRAF:14-3-3_2:MEK (PDB ID 7MFD) to corresponding cryo-EM density (EMD-23813)**
**Representative regions in the KD C-lobe**	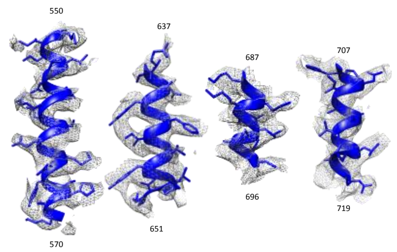	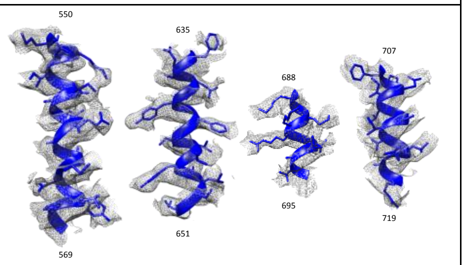
**αC- helix in "out" conformation**<br>The distance between the "in" and "out" position for the αC-helix is ~ 6-7 Å. The αC-helix fits well in the "out" position, as is observed in PDBs 6NYB (Park *et al*., 2019) and 6U2G (Liu *et al*., 2020).	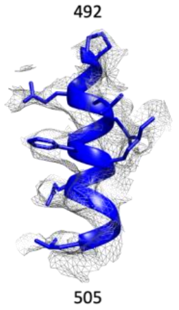	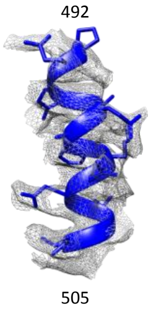
**Inhibitory turn**<br>(Residues A598-S602)<br>Similar to PDB 6NYB (Park *et al*., 2019), the inhibitory turn is observed.	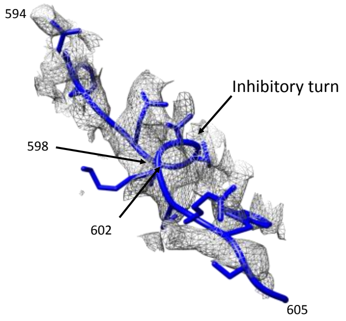	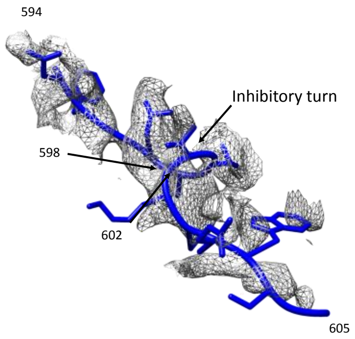

<table>
<tr>
<td>Broken R spine

(with L505 misaligned)

Similar to PDBs 6NYB (Park et al., 2019) and 6U2G (Liu et al., 2020), the R-spine is broken.</td>
<td>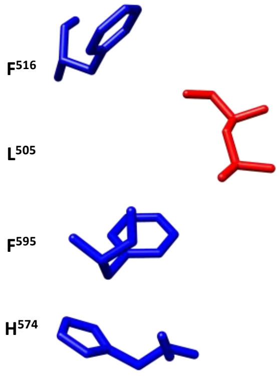

(Fit of L505 is shown above)</td>
<td>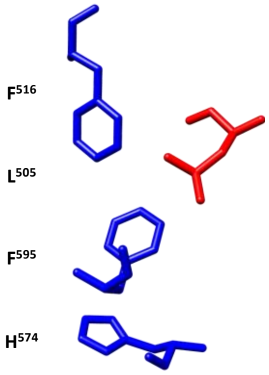

(Fit of L505 is shown above)</td>
</tr>
<tr>
<td>Active site region</td>
<td>BRAF:14-3-3$_2$ map (EMD-23814)
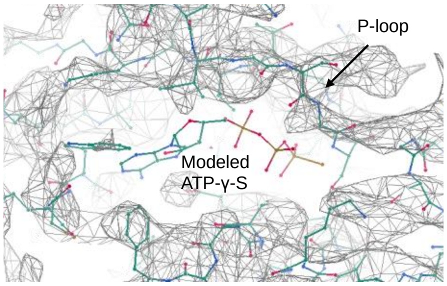
</td>
<td>BRAF:14-3-3$_2$:MEK map (EMD-23813)
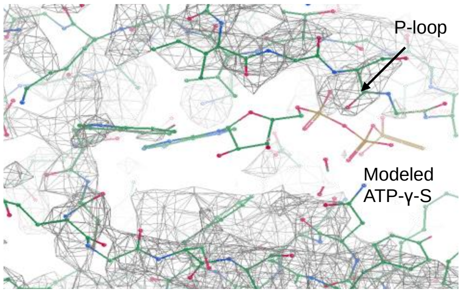
</td>
</tr>
</table>

Supplementary Table 2. Fitting of active and inactive BRAF KD conformations onto the KD density map of BRAF:14-3-3$_2$ and BRAF:14-3-3$_2$:MEK

BRAF KD active (PBD ID 2FB8) (left) and inactive (PDB ID 6U2G) (middle) structures fitted onto the BRAF:14-3-3$_2$ (EMD-23814) KD density map and compared with that in the BRAF:14-3-3$_2$ (PDB ID 7MFE) structure in this study (right)

Regions with unoccupied or misfitted densities are indicated by dashed lines. Two views with different orientations are shown.

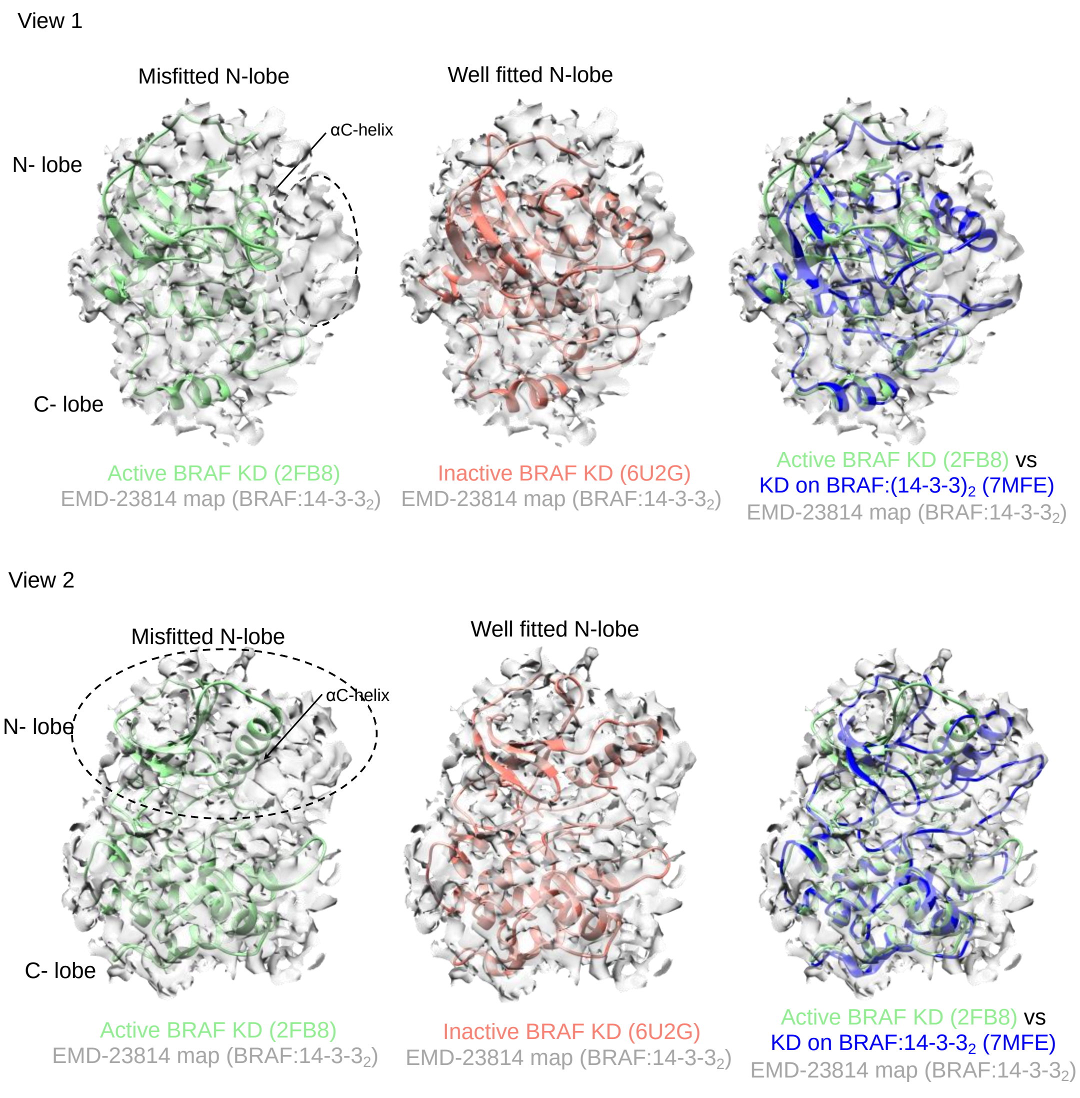

BRAF KD active (PBD ID 2FB8) (left) and inactive (PDB ID 6U2G) (middle) conformations fitted onto the BRAF:14-3-3_2:MEK (EMD-23813) map at the KD region and compared with that in the BRAF:14-3-3_2:MEK (PDB ID 7MFD) structure in this study (right)

Regions with unoccupied or misfitted densities are indicated by dashed lines. Two views with different orientations are shown.

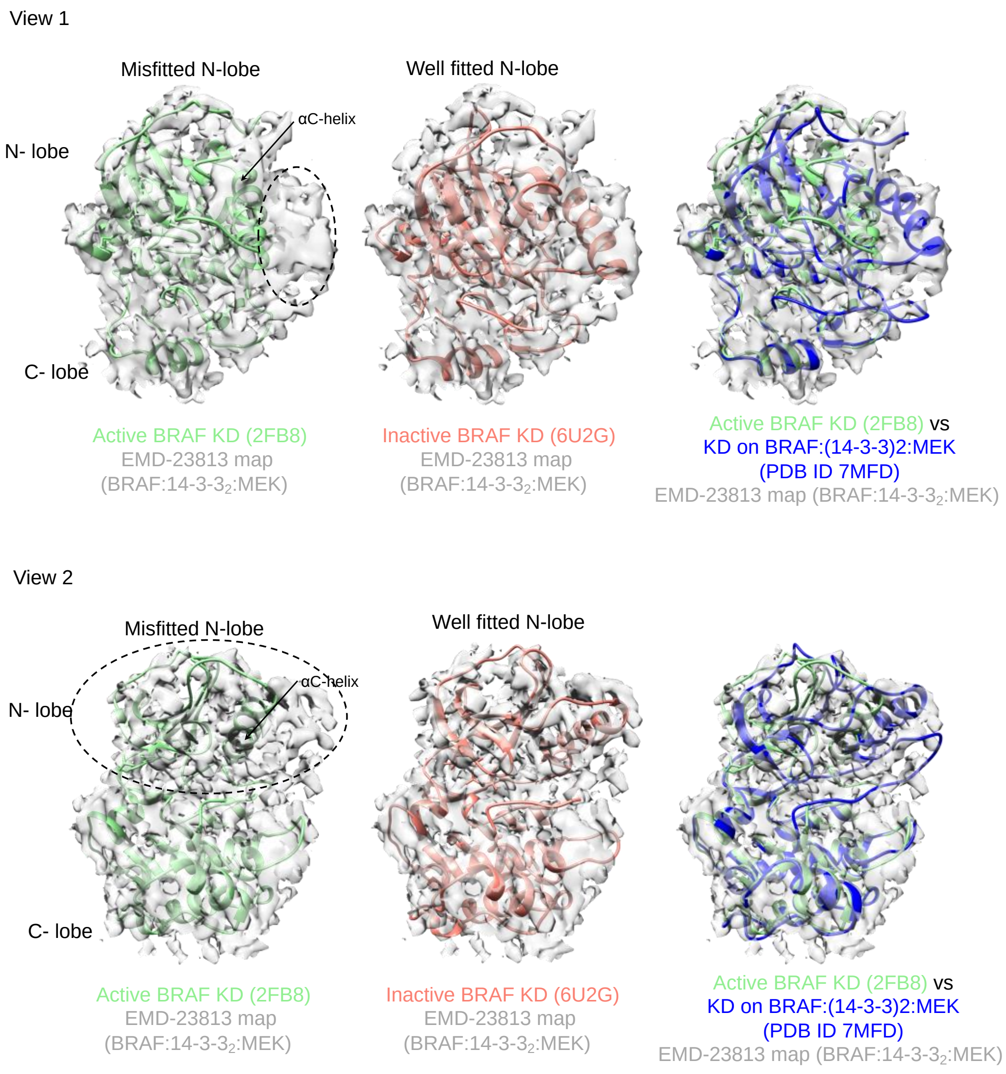

Orientation of the N-lobe relative to the C-lobe

Conformation is similar to that in PDBs 6NYB (Park *et al*., 2019) and 6U2G (Liu *et al*., 2020) with the N- and C- lobes exhibiting ~13° closer orientation than is observed in RAF-inhibitor-bound KDs (4RZV)

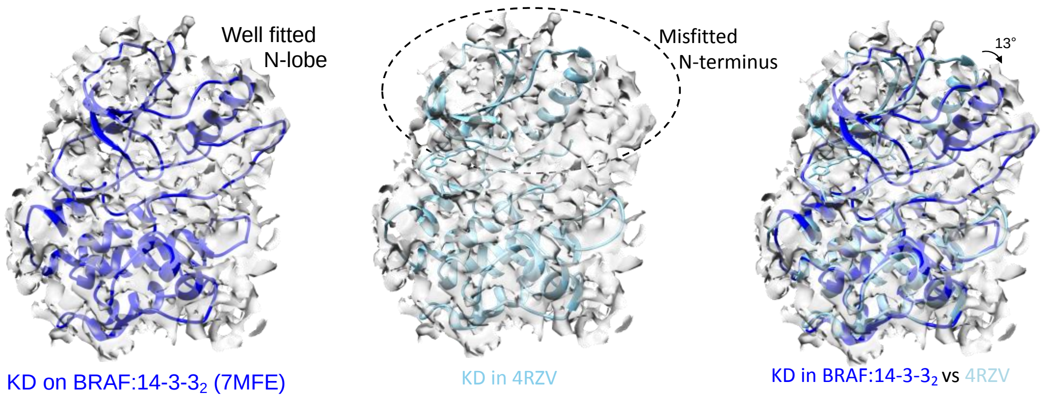

Supplementary Table 3. Reagent or Resource Table

REAGENT or RESOURCE	SOURCE	IDENTIFIER
**Antibodies**		
HaloTag mouse monoclonal	Promega	cat# G9211; RRID:AB_2688011
Rluc rabbit polyclonal	MBL International	cat# PM047; RRID:AB_1520866
GFP mouse monoclonal	Roche	cat# 11814460001; RRID:AB_390913
GFP rat monoclonal	MBL International	cat# D153-3; RRID:AB_591817
RAS10 mouse monoclonal	Millipore	cat# 05-516; RRID:AB_11211664
BRAF F-7 mouse monoclonal	Santa Cruz Biotechnology	cat# sc-5284; RRID:AB_626760
pS217/221-MEK rabbit polyclonal	Cell Signaling Technology	cat# 9121; RRID: AB_331648
MEK 1 mouse monoclonal	BD Biosciences	cat# 610122; RRID:AB_397528
MEK 2 mouse monoclonal	BD Biosciences	cat# 610236; RRID:AB_397631
**Critical Commercial Assays**		
Superose 6, 10/300 GL	Cytiva	cat# 29091596
Pierce Silver Stain Kit	Thermo Scientific	cat# 24612
NanoBRET Kit	Promega	cat# N1661
Halolink Resin	Promega	cat# G1915
Halo-TEV	Promega	cat# G6602
**Deposited Data**		
BRAF:14-3-3_2:MEK	This paper	Coordinates: PDBID: 7MFD<br>Cryo-EM map: EMDB: EMD-23813
BRAF:14-3-3_2	This paper	Coordinates: PDBID: 7MFE<br>Cryo-EM map: EMDB: EMD-23814
$BRAF_2$:14-3-3_2	This paper	Coordinates: PDBID: 7MFF<br>Cryo-EM map: EMDB: EMD-23815
Coordinates of RAF-1 cysteine-rich domain	(Moot et al., 1996)[1]	PDB ID: 1FAR
Coordinates of BRAF kinase domain bound to SB-590885	(King et al., 2006)[2]	PDB ID: 2FB8
Coordinates of Human MEK1 kinase in complex with CH5126766 and MgAMP-PNP	(Lito et al., 2014)[3]	PDB ID: 3WIG
Coordinates of 14-3-3 isoform zeta in complex with a diphoyphorylated CRAF peptide	(Molzan and Ottmann, 2012)[4]	PDB ID: 4FJ3
Coordinates of the BRAF:MEK1 complex	(Haling et al., 2016)[5]	PDB ID: 4MNE
Coordinates of BRAF (R509H) kinase domain monomer bound to Vemurafenib	(Karoulia et al., 2016)[6]	PDB ID: 4RZV
Coordinates of RAS Binding Domain (RBD) of BRAF	(Athuluri-Divakar et al., 2016)[7]	PDB ID: 5J17
Coordinates of BRAF:MEK1: 14-3-3	(Park et al., 2019)[8]	PDB ID: 6NYB
Coordinates of BRAF:14-3-3	(Kondo et al., 2019)[9]	PDB ID: 6UAN

dimer complex		
Coordinates of BRAF:MEK complex with AMP-PCP	(Liau et al., 2020)[10]	PDB ID: 6U2G
Coordinates of BRAF dimer bound to 14-3-3	(Liau et al., 2020)[10]	PDB ID: 6U2H
Coordinates of KRAS:CRAF_RBD-CRD complex	(Tran et al., 2021)[11]	PDB ID: 6XI7
Coordinates of RAS:RBD-CRD	(Cookis and Mattos, 2021)[12]	PDB ID: 7JHP
**Experimental Models: Cell lines**		
293FT (human)	ATCC	cat# PTA-5077, RRID:CVCL_6911
293T (human)	ATCC	cat# CRL-11268; RRID:CVCL_1926
HeLa (human, female)	ATCC	cat# CCL-2; RRID:CVCL_0030
Phoenix-Eco (human)	ATCC	cat# CRL-3214; RRID:CVCL_H717
NIH-3T3 (mouse)	ATCC	cat# CRL-1658; RRID:CVCL_0594
**Recombinant DNA**		
pCMV5-Halo-BRAF^{WT}	NCI-Ras Initiative	N/A
pCMV5-Venus-KRAS^{G12V}	NCI-Ras Initiative	N/A
pCMV5-Halo-KRAS^{G12V}	NCI-Ras Initiative	N/A
Lenti-puro-CMV-HA-KRAS^{G12V}	NCI-Ras Initiative	N/A
pLHCX-BRAF^{FL}-RLuc8	(Terrell et al., 2019)[13]	N/A
pLHCX-BRAF^{FL} R158A-RLuc8	This paper	N/A
pLHCX-BRAF^{FL} R166A-RLuc8	This paper	N/A
pLHCX-BRAF^{FL} K183A-RLuc8	This paper	N/A
pLHCX-BRAF^{FL} R188L-RLuc8	This paper	N/A
pLHCX-BRAF^{FL} M186A/M187A-RLuc8	This paper	N/A
pLHCX-BRAF^{FL} M186W/M187W-RLuc8	This paper	N/A
pLHCX-BRAF^{FL} M186K/M187V-RLuc8	This paper	N/A
pCMV5-BRAF^{REG}-Halo	This paper	N/A
pCMV5- BRAF^{REG} M186A/M187A -Halo	This paper	N/A
pCMV5- BRAF^{REG} M186W/M187W -Halo	This paper	N/A
pCMV5- BRAF^{REG} M186K/M187V -Halo	This paper	N/A
pCMV5- BRAF^{REG} T241P -Halo	This paper	N/A
pCMV5-NanoLuc-CRAF^{Cat}	This paper	N/A
pCMV5-BRAF^{RBD}-Halo	This paper	N/A
pCMV5- BRAF^{RBD} M186A/M187A -Halo	This paper	N/A
pCMV5- BRAF^{RBD} M186W/M187W -Halo	This paper	N/A
pCMV5- BRAF^{RBD}	This paper	N/A

M186K/M187V -Halo		
pBABE-puro-Flag-BRAF	(Terrell et al., 2019)[13]	N/A
pBABE-puro-Flag-BRAF M186A/M187A	This paper	N/A
pBABE-puro-Flag-BRAF M186W/M187W	This paper	N/A
pBABE-puro-Flag-BRAF T241P	This paper	N/A
**Software and Algorithms**		
Serial EM 3-7-6-64	(Mastronarde, 2005)[14]	http://bio3d.colorado.edu/SerialEM
Relion 3.1	(Zivanov et al., 2018) [15]	https://www3.mrc-lmb.cam.ac.uk/relion/index.php/Main_Page
MotionCor2	(Zheng et al., 2017) [16]	http://msg.ucsf.edu/em/software/motioncor2.html
Gctf	(Zhang, 2016) [17]	https://www2.mrc-lmb.cam.ac.uk/research/locally-developed-software/zhang-software/
COOT 0.9.6	(Emsley and Cowtan, 2004) [18]	https://www2.mrc-lmb.cam.ac.uk/personal/pemsley/coot
PHENIX 1.17.1	(Adams et al., 2010) [19]	https://www.phenix-online.org
Chimera 1.13.1	(Pettersen et al., 2004) [20]	https://www.cgl.ucsf.edu/chimera
Prism 8	N/A	https://www.graphpad.com/scientific-software/prism
**Other**		
R1.2/1.3 300 mesh Au holey carbon grids	Electron Microscopy Sciences	Cat# Q350AR1.3

Supplementary Table 4. Cryo-EM data collection, Refinement and Validation Statistics

	BRAF:14-3-3$_2$:MEK	BRAF:14-3-3$_2$	BRAF$_2$:14-3-3$_2$
**Data Processing**			
Data availability	PDB ID: 7MFD, EMB-23813	PDB ID: 7MFE, EMB-23814	PDB ID: 7MFF, EMB-23815
Magnification	130k, EFTEM mode	105k, EFTEM mode	105k, EFTEM mode
Voltage (kV)	300	300	300
Electron exposure (e^-/Å^2)	57	55	55
Exposure Rate (e^-/Å^2/s)	7.13	5.5	5.5
Dose/frame (e^-/Å^2)	1.14	1.10	1.10
**Collection**			
Number of frames collected per micrograph	50	50	50
Energy filter slit width	20eV	20eV	20eV
Defocus range (μm)	-0.8 to -2.5	-0.8 to -2.5	-0.8 to -2.5
Pixel size (Å)	1.058	1.348	1.348
Symmetry imposed	C1	C1	C1
Movies (no.)	3,976	5995	2,998
Initial particle images (no.)	1,824,538	2,496,302	2,159,824
Final particle images (no.)	142,852	198,731	203,343
Map resolution (Å)	3.66	4.07	3.89
FSC threshold	0.143		
**Refinement**			
Initial model used (PDB ID)	1FAR, 3WIG, 4FJ3, 4MNE, 5J17	1FAR, 4FJ3, 4MNE, 5J17	2FB8, 4FJ3
Map sharpening B factor (Å^2)	-100.5	-168.6	-167.4
Model composition			
Non-hydrogen atoms	9127	6843	8008
Protein residues	1147	859	1000
Ligands	1	0	2
Metals	2	2	0
B factors (Å^2)			
Protein	80.3	35.9	41.8
Ligand	94.6	42.2	23.6
r.m.s deviations			
Bond lengths (Å)	0.004	0.002	0.002
Bond angles (°)	0.659	0.570	0.522
**Validation statistics**			

MolProbity score	2.19	2.18	1.69
Clash Score	17.6	17.8	8.6
Rotamer outliers (%)	0	0	0.12
Ramachandran Plot			
Favored (%)	93.0	93.4	96.5
Allowed (%)	7.0	6.6	3.5
Outliers (%)	0	0	0
Model vs Data			
CC (mask)	0.61	0.64	0.71
CC (box)	0.68	0.63	0.69
CC (peaks)	0.53	0.50	0.61
CC (volume)	0.60	0.62	0.69
Mean CC for ligands	0.69	0.72	0.71

Supplementary References

1 Mott, H. R. *et al.* The solution structure of the Raf-1 cysteine-rich domain: a novel ras and phospholipid binding site. *Proceedings of the National Academy of Sciences of the United States of America* **93**, 8312-8317 (1996).

2 King, A. J. *et al.* Demonstration of a genetic therapeutic index for tumors expressing oncogenic BRAF by the kinase inhibitor SB-590885. *Cancer Res* **66**, 11100-11105, doi:10.1158/0008-5472.Can-06-2554 (2006).

3 Lito, P. *et al.* Disruption of CRAF-mediated MEK activation is required for effective MEK inhibition in KRAS mutant tumors. *Cancer Cell* **25**, 697-710, doi:10.1016/j.ccr.2014.03.011 (2014).

4 Molzan, M. & Ottmann, C. Synergistic binding of the phosphorylated S233- and S259-binding sites of C-RAF to one 14-3-3ζ dimer. *J Mol Biol* **423**, 486-495, doi:10.1016/j.jmb.2012.08.009 (2012).

5 Haling, J. R. *et al.* Structure of the BRAF-MEK complex reveals a kinase activity independent role for BRAF in MAPK signaling. *Cancer Cell* **26**, 402-413, doi:10.1016/j.ccr.2014.07.007 (2014).

6 Karoulia, Z. *et al.* An Integrated Model of RAF Inhibitor Action Predicts Inhibitor Activity against Oncogenic BRAF Signaling. *Cancer Cell* **30**, 485-498, doi:10.1016/j.ccell.2016.06.024 (2016).

7 Athuluri-Divakar, S. K. *et al.* A Small Molecule RAS-Mimetic Disrupts RAS Association with Effector Proteins to Block Signaling. *Cell* **165**, 643-655, doi:10.1016/j.cell.2016.03.045 (2016).

8 Park, E. *et al.* Architecture of autoinhibited and active BRAF-MEK1-14-3-3 complexes. *Nature*, doi:10.1038/s41586-019-1660-y (2019).

9 Kondo, Y. *et al.* Cryo-EM structure of a dimeric B-Raf:14-3-3 complex reveals asymmetry in the active sites of B-Raf kinases. *Science* **366**, 109-115, doi:10.1126/science.aay0543 (2019).

10 Liau, N. P. D. *et al.* Negative regulation of RAF kinase activity by ATP is overcome by 14-3-3-induced dimerization. *Nat Struct Mol Biol* **27**, 134-141, doi:10.1038/s41594-019-0365-0 (2020).
11 Tran, T. H. *et al.* KRAS interaction with RAF1 RAS-binding domain and cysteine-rich domain provides insights into RAS-mediated RAF activation. *Nat Commun* **12**, 1176, doi:10.1038/s41467-021-21422-x (2021).
12 Cookis, T. & Mattos, C. Crystal structure reveals the full Ras-Raf interface and advances mechanistic understanding of Raf activation. *Biomolecules* **11**, 996, doi:10.3390/biom11070996 (2021).
13 Terrell, E. M. *et al.* Distinct Binding Preferences between Ras and Raf Family Members and the Impact on Oncogenic Ras Signaling. *Mol Cell* **76**, 872-884 e875, doi:10.1016/j.molcel.2019.09.004 (2019).
14 Mastronarde, D. N. Automated electron microscope tomography using robust prediction of specimen movements. *J Struct Biol* **152**, 36-51, doi:10.1016/j.jsb.2005.07.007 (2005).
15 Zivanov, J. *et al.* New tools for automated high-resolution cryo-EM structure determination in RELION-3. *Elife* **7**, doi:10.7554/eLife.42166 (2018).
16 Zheng, S. Q. *et al.* MotionCor2: anisotropic correction of beam-induced motion for improved cryo-electron microscopy. *Nat Methods* **14**, 331-332, doi:10.1038/nmeth.4193 (2017).
17 Zhang, K. Gctf: Real-time CTF determination and correction. *J Struct Biol* **193**, 1-12, doi:10.1016/j.jsb.2015.11.003 (2016).
18 Emsley, P. & Cowtan, K. Coot: model-building tools for molecular graphics. *Acta Crystallogr D Biol Crystallogr* **60**, 2126-2132, doi:10.1107/s0907444904019158 (2004).
19 Adams, P. D. *et al.* PHENIX: a comprehensive Python-based system for macromolecular structure solution. *Acta Crystallogr D Biol Crystallogr* **66**, 213-221, doi:10.1107/s0907444909052925 (2010).
20 Pettersen, E. F. *et al.* UCSF Chimera--a visualization system for exploratory research and analysis. *J Comput Chem* **25**, 1605-1612, doi:10.1002/jcc.20084 (2004).
