# Supplementary material for: Structural insights into the BRAF monomer-to-dimer transition mediated by RAS binding
Source: Nat Commun. 2022 Jan 25;13:486. doi: 10.1038/s41467-022-28084-3 (PMC8789793; doi:10.1038/s41467-022-28084-3)
Supplement: Supplementary file 3 — Reporting Summary [file 41467_2022_28084_MOESM3_ESM.pdf]

nature portfolio

Corresponding author(s): Morrison, Zhang

Last updated by author(s): January 04, 2021

Reporting Summary

Nature Portfolio wishes to improve the reproducibility of the work that we publish. This form provides structure for consistency and transparency in reporting. For further information on Nature Portfolio policies, see our Editorial Policies and the Editorial Policy Checklist.

Statistics

For all statistical analyses, confirm that the following items are present in the figure legend, table legend, main text, or Methods section.

n/a	Confirmed	
☐	☒	The exact sample size (*n*) for each experimental group/condition, given as a discrete number and unit of measurement
☐	☒	A statement on whether measurements were taken from distinct samples or whether the same sample was measured repeatedly
☒	☐	The statistical test(s) used AND whether they are one- or two-sided<br>*Only common tests should be described solely by name; describe more complex techniques in the Methods section.*
☒	☐	A description of all covariates tested
☐	☒	A description of any assumptions or corrections, such as tests of normality and adjustment for multiple comparisons
☐	☒	A full description of the statistical parameters including central tendency (e.g. means) or other basic estimates (e.g. regression coefficient) AND variation (e.g. standard deviation) or associated estimates of uncertainty (e.g. confidence intervals)
☒	☐	For null hypothesis testing, the test statistic (e.g. *F*, *t*, *r*) with confidence intervals, effect sizes, degrees of freedom and *P* value noted<br>*Give P values as exact values whenever suitable.*
☒	☐	For Bayesian analysis, information on the choice of priors and Markov chain Monte Carlo settings
☒	☐	For hierarchical and complex designs, identification of the appropriate level for tests and full reporting of outcomes
☒	☐	Estimates of effect sizes (e.g. Cohen's *d*, Pearson's *r*), indicating how they were calculated

Our web collection on statistics for biologists contains articles on many of the points above.

Software and code

Policy information about availability of computer code

Data collection	Serial EM 3-7-6-64 was used for cryo-EM data collection
Data analysis	Relion 3.1, MotionCor2 1.3.0, Gctf 1.0.6, COOT 0.9.6, PHENIX 1.17.1, Chimera 1.13.1, were used for the analysis of cryo-EM data and generation of structural figures.<br>Prism 8 was used for the analysis of Fluorescence polarization and BRET assays

For manuscripts utilizing custom algorithms or software that are central to the research but not yet described in published literature, software must be made available to editors and reviewers. We strongly encourage code deposition in a community repository (e.g. GitHub). See the Nature Portfolio guidelines for submitting code & software for further information.

Data

Policy information about availability of data

All manuscripts must include a data availability statement. This statement should provide the following information, where applicable:

- Accession codes, unique identifiers, or web links for publicly available datasets
- A description of any restrictions on data availability
- For clinical datasets or third party data, please ensure that the statement adheres to our policy

Three-dimensional cryo-EM density maps have been deposited in the Electron Microscopy data Bank under accession numbers EMD-23813 (BRAF:14-3-32:MEK) [https://www.ebi.ac.uk/emdb/EMD-23813], EMD-23814 (BRAF:14-3-32) [https://www.ebi.ac.uk/emdb/EMD-23814], and EMD-23815 (BRAF2:14-3-32) [https://www.ebi.ac.uk/emdb/EMD-23815]. The coordinates of atomic models have been deposited in the Protein Data Bank under accession numbers PDB ID: 7MFD (BRAF:14-3-32:MEK) [https://doi.org/10.2210/pdb7MFD/pdb], 7MFE (BRAF:14-3-32) [https://doi.org/10.2210/pdb7MFE/pdb] and 7MFF (BRAF2:14-3-32) [https://doi.org/10.2210/pdb7MFF/pdb]. Previously published accession number PDB IDs used in this manuscript are: 1FAR [http://doi.org/10.2210/pdb1FAR/pdb], 2FB8

[http://doi.org/10.2210/pdb2FB8/pdb], 3WIG [http://doi.org/10.2210/pdb3WIG/pdb], 4FJ3 [http://doi.org/10.2210/pdb4FJ3/pdb], 4MNE [http://doi.org/10.2210/pdb4MNE/pdb], 4RZV [http://doi.org/10.2210/pdb4RZV/pdb], 5J17 [http://doi.org/10.2210/pdb5J17/pdb], 6NYB [http://doi.org/10.2210/pdb6NYB/pdb], 6UAN [http://doi.org/10.2210/pdb6UAN/pdb], 6U2G [http://doi.org/10.2210/pdb6U2G/pdb], 6U2H [http://doi.org/10.2210/pdb6U2H/pdb], 6XI7 [http://doi.org/10.2210/pdb6XI7/pdb], 7JHP [http://doi.org/10.2210/pdb7JHP/pdb]. Source data are provided with this paper.

Field-specific reporting

Please select the one below that is the best fit for your research. If you are not sure, read the appropriate sections before making your selection.

- [x] Life sciences
- [ ] Behavioural & social sciences
- [ ] Ecological, evolutionary & environmental sciences

For a reference copy of the document with all sections, see nature.com/documents/nr-reporting-summary-flat.pdf

Life sciences study design

All studies must disclose on these points even when the disclosure is negative.

Sample size	Biochemical and cellular data were from at least duplicate independent experiments. The sample sizes used were sufficient to represent the reproducibility of the assays.
Data exclusions	There were no data exclusions.
Replication	The biochemical and cellular experiments were reproducible. The number of replications can be found in the figure legends and Data source file.
Randomization	It was unnecessary to perform randomized experiments in the current study.
Blinding	It was unnecessary to perform blinded experiments in the current study.

Reporting for specific materials, systems and methods

We require information from authors about some types of materials, experimental systems and methods used in many studies. Here, indicate whether each material, system or method listed is relevant to your study. If you are not sure if a list item applies to your research, read the appropriate section before selecting a response.

Materials & experimental systems

n/a	Involved in the study	
[ ]	[x]	Antibodies
[ ]	[x]	Eukaryotic cell lines
[x]	[ ]	Palaeontology and archaeology
[x]	[ ]	Animals and other organisms
[x]	[ ]	Human research participants
[x]	[ ]	Clinical data
[x]	[ ]	Dual use research of concern

Methods

n/a	Involved in the study	
[x]	[ ]	ChIP-seq
[x]	[ ]	Flow cytometry
[x]	[ ]	MRI-based neuroimaging

Antibodies

Antibodies used	HaloTag – Promega #G9211 (1:1000); Rluc – MBL #PM047 (1:1000); GFP mouse – Roche #11814460001 (1:1000); GFP rat clone RQ2 – MBL # D153-3 (2uL/ml for IP); RAS10 – Millipore #05-516; BRAF F-7 – SCBT # sc-5284 (1:1000); pS217/221-MEK – Cell Signaling #9121 (1:1000); MEK1 – BD #610122 (1:5000); MEK2 – BD #610236 (1:5000)
Validation	Validation of the antibodies in terms of species recognition and application was obtained from the supplier's data sheets. The supplier and RRID numbers for the antibodies are listed in Supplementary Table 3. HaloTag antibody recognizes HaloTag fusion proteins in immunoblot analysis. The Rluc antibody can be used for immunoblotting, immunoprecipitation, and immunocytochemistry, and it does not cross react with firefly luciferase. GFP mouse antibody can be used for immunoblotting, immunoprecipitation, and immunostaining. GFP rat antibody clone RQ2 recognizes EBFP, ECFP, EGFP, Venus, and Sapphire, and it can be used for immunoblotting, immunoprecipitation, and immunocytochemistry, immunohistochemistry, and flow cytometry. GFP Rat Clone RQ2 RAS10 antibody detects H-, K-, and N-RAS proteins by ELISA, flow cytometry, immunocytochemistry, immunohistochemistry, immunoprecipitation, and immunoblotting, RAS10 recognizes RAS proteins of human, mouse, and rat origin. BRAF F-7 antibody was raised against amino acids 12-156 of human BRAF and can detect endogenous or recombinant BRAF protein by immunoblotting, immunoprecipitation, immunofluorescence, immunohistochemistry, and ELISA. BRAF F-7 recognizes BRAF protein of mouse, rat, and human origin. pS217/221-MEK antibody detects endogenous levels of MEK1/2 when activated by phosphorylation at Ser217/221. This antibody does not cross-react with related kinases such as activated SEK (MKK4), MKK3 or MKK6, but will react with MEK1/2 singly phosphorylated at Ser217 or singly phosphorylated at Ser221. The pS217/221-MEK antibody can be used for immunoblotting, immunoprecipitation, immunohistochemistry, chromatin immunoprecipitation, immunofluorescence, flow cytometry, and ELISA, and it recognizes phosphorylated MEK proteins of human, mouse, rat, and monkey

origin. MEK1 antibody was raised against full-length, human MEK1 and detects endogenous levels of MEK1. The MEK1 antibody can be used for immunoblotting, immunoprecipitation, immunohistochemistry, and bioimaging. This antibody recognizes human MEK1 and may also react to chicken, dog, frog, mouse, and rat MEK1. MEK2 antibody was raised against full-length rat MEK2 and detects endogenous levels of MEK2. The MEK2 antibody can be used for immunoblotting, immunoprecipitation, immunofluorescence, and immunohistochemistry. This antibody recognizes human, chicken, dog, frog, mouse, and rat MEK2.

Eukaryotic cell lines

Policy information about cell lines

Cell line source(s)	HEK-293FT, HEK-293T, Hela, Phoenix-Eco, and NIH-3T3 cells were obtained from ATCC.
Authentication	Cell lines were obtained from ATCC and not independently authenticated.
Mycoplasma contamination	All cell lines tested negative for mycoplasma.
Commonly misidentified lines (See ICLAC register)	No misidentified lines were used.
